# Role of Endothelial Cell Lipoprotein Lipase for Brown Adipose Tissue Lipid and Glucose Handling

**DOI:** 10.3389/fphys.2022.859671

**Published:** 2022-03-29

**Authors:** Ellen Thiemann, Gerburg K. Schwaerzer, Ioannis Evangelakos, Marceline M. Fuh, Michelle Y. Jaeckstein, Janina Behrens, Stefan K. Nilsson, Manju Kumari, Ludger Scheja, Alexander Pfeifer, Joerg Heeren, Markus Heine

**Affiliations:** ^1^ Department of Biochemistry and Molecular Cell Biology, University Medical Center Hamburg-Eppendorf, Hamburg, Germany; ^2^ Institute of Pharmacology and Toxicology, University Hospital, University of Bonn, Bonn, Germany; ^3^ Department of Medical Biosciences/Physiological Chemistry, Umeå University, Umeå, Sweden; ^4^ Department of Internal Medicine III, Heidelberg University, Heidelberg, Germany

**Keywords:** lipoprotein lipase, triglycerides, endothelial cells, adipocytes, lipoproteins, adipose tissue, thermogenesis, de novo lipogenesis

## Abstract

Cold-induced activation of brown adipose tissue (BAT) has an important impact on systemic lipoprotein metabolism by accelerating the processing of circulating triglyceride-rich lipoproteins (TRL). Lipoprotein lipase (LPL) expressed by adipocytes is translocated via endothelial to the capillary lumen, where LPL acts as the central enzyme for the vascular lipoprotein processing. Based on preliminary data showing that LPL is not only expressed in adipocytes but also in endothelial cells of cold-activated BAT, we aimed to dissect the relevance of endothelial versus adipocyte LPL for lipid and energy metabolism in the context of adaptive thermogenesis. By metabolic studies we found that cold-induced triglyceride uptake into BAT, lipoprotein disposal, glucose uptake and adaptive thermogenesis were not impaired in mice lacking *Lpl* exclusively in endothelial cells. This finding may be explained by a compensatory upregulation in the expression of adipocyte-derived *Lpl and endothelial lipase (Lipg)*.

## Introduction

Brown adipose tissue (BAT) is a thermogenically active organ of mammals that supports adaptation to cold environments through non-shivering thermogenesis. Heat is generated in active brown adipocytes through disconnection of the respiratory chain from oxidative phosphorylation by the proton transporter uncoupling protein-1 (UCP1) at the inner mitochondrial membrane (Cannon and Nedergaard, 2004). This process is highly connected with ß-oxidation of fatty acids that are released from intracellular lipid droplets. Lipolysis of stored triglycerides is initiated by sympathetic stimulation of ß-adrenergic receptors on brown adipocytes which initiates cyclic AMP (cAMP) signaling, resulting in activation of adipose triglyceride lipase and hormone-sensitive lipase (Young and Zechner, 2013). In rodents, BAT thermogenesis is mainly mediated by ß3-adrenergic receptor stimulation (Cannon and Nedergaard, 2004), whereas for human BAT evidence has been provided for both ß2-and ß3-adrenergic receptor signaling (Blondin et al., 2020; Cero et al., 2021). As activated BAT utilizes large amounts of fatty acids, efficient mechanisms are needed to replenish intracellular lipid stores. BAT is a highly vascularized tissue, and upon cold exposure, glucose (Bartelt et al., 2011; Stanford et al., 2013; Heine et al., 2018; Fischer et al., 2019) and fatty acids (Furler et al., 2000; Heine et al., 2018) are taken up in large quantities into brown adipocytes via the endothelium. Glucose handling by activated brown adipocytes is quite complex and a recent elegant *in vivo* study employing ^13^C-labeleled glucose showed that under acute cold exposure, glucose is primarily used as fuel for thermogenesis and for the pentose phosphate pathway (Jung et al., 2021). In response to sustained cold adaptation, ^13^C is also enriched in lactate and glycerol 3-phosphate (Jung et al., 2021). Moreover, under this condition glucose is also used for *de novo* lipogenesis (DNL), which confirms previous studies demonstrating DNL to be a highly active pathway in BAT (Mottillo et al., 2014; Weir et al., 2018). Non-esterified fatty acids (NEFA) are taken up via transporters such as CD36 and are either directly channeled into ß-oxidation or stored in the form of triglycerides in lipid droplets. These fatty acids are mostly derived from triglyceride-rich lipoproteins (TRL) which are processed by lipoprotein lipase (LPL) in the capillary lumen (Bartelt et al., 2011). LPL is the key enzyme for TRL degradation and is highly expressed in organs that consume or store fatty acids in large amounts such as heart, skeletal muscle, white adipose tissue (WAT), and BAT (Merkel et al., 2002; Bartelt et al., 2011; Kersten, 2014; Khedoe et al., 2015). In BAT, LPL is known to be predominantly expressed by brown adipocytes and is translocated to the luminal site of the vascular endothelium by glycosylphosphatidylinositol anchored high density lipoprotein binding protein 1 (GPIHBP1) (Beigneux et al., 2007; Davies et al., 2010; Davies et al., 2012; Olivecrona, 2016). The expression and activity of LPL is regulated in a tissue-specific manner and controlled by different stimuli to provide the optimal supply of organs with fatty acids (Olivecrona et al., 1997; Kersten, 2014). LPL in BAT is mainly stimulated by cold-induction but also refeeding and insulin administration can stimulate enzyme activity (Mitchell et al., 1992; Deshaies et al., 1993; Klingenspor et al., 1996; Kuusela et al., 1997). On the transcriptional level, adipocyte LPL expression is regulated by several transcription factors including among others the peroxisome proliferator-activated receptor gamma (Kersten, 2014). The importance of LPL for systemic lipid metabolism is demonstrated by the observation that LPL deficiency in humans and mice leads to massive hypertriglyceridemia (Coleman et al., 1995; Weinstock et al., 1995). In line, *Gpihbp1* ^−/−^ mice are characterized by highly increased plasma triglyceride levels due to the missing translocation of LPL. In the present study, we show that LPL is not only expressed in adipocytes but surprisingly also in endothelial cells of cold-activated BAT. Hence, we investigated the role of LPL expression in these cells by studying transgenic mice lacking LpL exclusively in endothelial cells (EndoLPLko). We observed that LPL expressed by endothelial cells is dispensable for lipoprotein handling and adaptive thermogenesis in both fasted and postprandial state. Furthermore, we provide evidence that the lack of effect on lipoprotein processing in EndoLPLko mice may be explained by a compensatory, higher expression of LPL in brown adipocytes and of LIPG in endothelial cells.

## Materials and Methods

### Animals

All experiments were performed with permission of the Animal Welfare Officers at University Medical Center Hamburg-Eppendorf and Behörde für Gesundheit und Verbraucherschutz Hamburg. To induce Cre-loxP recombination, Cdh5-Cre/ERT2 x *Lpl* ^flox/flox^ (EndoLPLko) mice received three consecutive doses of 0.2 mg tamoxifen (Sigma-Aldrich. St. Louis, Missouri, USA) dissolved in 100 µl sunflower oil via gavage 1 week before the experiments. All mice had ad libitum access to food and water and were kept in a temperature-controlled room at given temperatures with a 12 h light: 12 h dark cycle. The mice were fed a chow diet (P1324, Altromin, Germany) or western type diet, ssniff Spezialdiäten GmbH, Germany, duration: 2 weeks before necropsy), as indicated in the text. For the experiments, age- and weight-matched male mice were used. For the combined oral glucose and fat tolerance test (OGFT), turnover study and indirect calorimetry, mice were first fed chow diet and then received western-type diet for 2 weeks. For OGFT and lipoprotein turnover studies body composition (fat and lean mass) was analyzed using a magnetic whole-body composition analyzer (EchoMRI™, Zinsser Analytic GmbH, Eschborn, Germany) 1 day prior to the experiments. For all terminal procedures, mice received 180 mg/kg ketamine and 24 mg/kg xylazine before necropsy.

### Metabolic Tracer Studies

For OGFT, mice were fasted for 2 h before receiving an oral gavage of 200 µl of a glucose-lipid emulsion containing 47 mg triglycerides/kg body weight and glucose (2 g/kg body weight). The gavage solution was labelled with ^14^C-triolein (0.15 MBq/kg body weight, Perkin Elmer, Waltham, MA, USA) and ^3^H-deoxyglucose (^3^H-DOG; 0.72 MBq/kg body weight, Hartmann Analytic, Braunschweig, Germany). Organs were harvested 2 h after gavage. For lipoprotein turnover studies, mice were fasted for 4 h and were subsequently tail vein-injected with 100 µl radiolabeled TRLs. Radiolabeled TRLs were prepared by extraction of rat chylomicrons (Skottova et al., 1995) using the method of Folch, addition of radiolabelled ^14^C-triolein and subsequent sonication. Solvents were evaporated, and labelled TRL were formed by addition of PBS and ^14^C-triolein (MBq/kg body weight) and subsequent sonication. ^3^H-DOG (MBq/kg body weight) was added to the emulsion to follow glucose uptake from the circulation without triggering glucose-stimulated insulin secretion. Organs were harvested 15 min after injection. For all experiments, organs from anesthetized mice were harvested after systemic perfusion with PBS-heparin (10 U/mL; Rotexmedica) via the left heart ventricle. Organs were homogenized using Solvable™ (Perkin Elmer) or frozen immediately in liquid nitrogen and stored at −80°C for further analysis. Radioactive counts were determined by scintillation counting using a Tricarb scintillation counter (Liquid Scintillation Analyzer Tri-Carb^®^2810TR, Perkin Elmer).

For imaging of TRL disposal in BAT by confocal fluorescence microscopy, an immunofluorescence-based method was used. Briefly, BODIPY™ FL C 16 labeled TRL (BODIPY-TRL) were prepared using a similar approach to radiolabeled TRL. Briefly, 0.2 mg BODIPY™ FL C 16 (D3821, Thermofischer) dissolved in 1 ml Intralipid^®^ (CLINOLEIC 20%, Baxter S.A.) was applied intragastrically in rats and chylomicrons were obtained from cannulated lymphatic vessels. Lipids were extracted to obtain TRL particles as described above.

### TRAP RNA Isolation

TRAP was performed as previously described (Long et al., 2014) with modifications. In brief, small pieces (50–100 mg) of frozen BAT were Dounce-homogenized in 4 ml homogenization buffer (50 mM Tris [pH 7.5], 12 mM MgCl2, 100 mM KCl, 1% NP-40, 100 μg/ml Cycloheximide, 1 mg/ml sodium heparin, 2 mM DTT, 0.2 U/μL RNasin, and 1x Complete EDTA-free protease inhibitor; Roche). After centrifugation at 13,000 rpm for 10 min, the lipid layer was removed and the supernatant was collected and incubated with anti-GFP antibody (5 μg/ml; Abcam, ab290) for 1 h at 4°C. Protein G dynabeads were washed twice in low-salt wash buffer (50 mM Tris [pH 7.5], 12 mM MgCl2, 100 mM KCl, 1% NP-40, 100 μg/ml cycloheximide, and 2 mM DTT), added to the homogenates with antibody, and subsequently incubated for 30 min. Dynabeads with immunoprecipitates were washed three times in high-salt wash buffer (50 mM Tris [pH 7.5], 12 mM MgCl2, 300 mM KCl, 1% NP-40, 100 μg/ml cycloheximide, and 2 mM DTT). Following the last wash, RLT buffer with β-mercaptoethanol was added to dynabeads, and RNA was extracted using a QIAGEN Micro RNeasy kit according to the manufacturer’s instructions. For input RNA, 5% of homogenates were mixed with TRIzol and processed according to the manufacturer’s instructions to extract total RNA. Isolated RNA was quantified by Qubit.

### Gene Expression Analysis

To obtain SVF, interscapular BAT was minced and then digested with 1 mg/ml type II collagenase for 30 min at 37°C (Sigma Aldrich). The dissociated cells were passed through a 100 μm sieve to remove undigested particles. Centrifugation at 700 *g* for 10 min was then performed to separate the SVF pellet from the floating adipocytes. The resulting SVF pellet was dissolved in PBS and passed through a 40 µm sieve to achieve higher purity. For isolation of endothelial cells and brown adipocytes, the filtrate was centrifuged at 600 x g for 5 min, the cell pellet was resuspended and incubated with CD11b MicroBeads for depletion of the macrophage fraction (Miltenyi; 10 µl beads/107 cells). CD11b + cells were captured from the lysate using magnetic columns (Miltenyi). The flow through was centrifuged, the pellet was resuspended and incubated with CD31 MicroBeads (Miltenyi; 10 µl beads/107 cells) to isolate endothelial cells. The flow through, containing predominantly adipocytes, was collected. RNA was isolated from cells, tissue samples and SVF using TRIzol Reagent (ThermoFischer Scientific, Waltham, MA, USA) and NucleoSpin RNA/Protein kit (Macherey & Nagel, Düren, Germany) and used for cDNA preparation using the High Capacity cDNA Reverse Transcription kit with RNase Inhibitor (ThermoFischer Scientific) according to the manufacturer’s instructions. Real-time PCR using TaqMan Assay-on-Demand primer sets was performed on a QuantStudio 5 Real-Time-PCR System (ThermoFischer Scientific) and relative expression was normalized to the housekeeper Tbp. Taqman^®^ assays used in this study (assay IDs in brackets): *Acaca* (Mm01304285_m1), *Angptl4* (Mm00480431_m1), *Cd36* (Mm00432403_m1), *Chrebpß* (AIVI4CH), *Dio2* (Mm00515664_m1), *Elovl3* (Mm00468164_m1), *Elovl6* (Mm00851223_s1), *Fasn* (Mm00662319_m1), *Glut4* (Mm01245502_m1), *Lipg* (Mm00495368_m1), *Ppargc1a* (Mm00447183_m1), *Scd1* (Mm00772290_m1), *Srebp1c* (AI89KJW), *Tbp* (Mm00446973_m1), U*cp1* (Mm00494069_m1).

### Plasma and Lipid Parameters

Plasma triglyceride (Triglyceride FS Kit, DiaSys, Holzheim, Germany), cholesterol (Cholesterin FS Kit, DiaSys), and NEFA levels (NEFA-HR (2)-Kit, FUJIFILM Wako Chemicals, Neuss, Germany) were determined using commercial kits according to the manufacturer’s instructions. Blood glucose was determined by conventional test stripes (Accu-Chek, Roche).

### Histology and Adipocyte Diameter Determination

A 5 µm thick hematoxylin and eosin-stained section were used for microscopy and size determination. The adipocyte diameter estimates were calculated by NIS-Elements from BAT of 5 independent mice per group.

### Indirect Calorimetry and Body Core Temperature

Indirect calorimetry was performed in a TSE phenomaster (TSE Systems) in a temperature- and humidity-controlled chamber as described before (Heine et al., 2018). During the experiment, all mice were housed in single cages at a 12 h light: 12 h dark cycle and had ad libitum access to food and water. EndoLPLko and control mice were kept in the system at 22°C for 6d and for the analysis of energy expenditure, the temperature was decreased to 6°C for 7d. Oxygen consumption and carbon dioxide production were measured every 15 min. Body core temperature was continuously determined using an implantable mouse telemetry system based on G2 E- Mitter (Minimitter System by Starr Life Science) in the TSE phenomaster.

### Transwell System

Murine brown adipocytes were seeded and differentiated in the lower chamber of a transwell system. On day seven of the brown adipocyte differentiation protocol (Haas et al., 2009), murine microvascular endothelial cells (InSCREENex GmbH) seeded in a 0.4 µM pore size TC-insert (Sarstedt) were added on the top of the adipocytes. Both brown adipocytes and endothelial cells were (co-)cultured for 4 hours in a media containing 50% differentiation media (DMEM Glutamax (ThermoFisher Scientific) supplemented with 10% FBS, 100 IU/ml penicillin, 100 μg/ml streptomycin, 20 nM insulin, 1 nM triiodothyronine) and 50% endothelial cell media (InSCREENex GmbH) with or without 10 µM CL316,243. RNA was isolated from brown adipocytes and endothelial cells separately.

### Statistical Analyses

Data are presented as mean ± SEM. Two groups were compared by unpaired two-tailed Student’s t test, more than two groups by one-way or two-way ANOVA, as indicated in the figure legends. No method was used to determine whether the data met assumptions of either Student’s t test or ANOVA. The statistical parameters (i.e., *p* values, numbers of biological repeats) can be found in the figure legends. No exclusion or inclusion criteria were used for data analyses. Statistical analyses were conducted using Graph Pad software; *p* < 0.05 was considered significant.

## Results

### Endothelial Cells in Murine Brown Adipose Tissue Express LPL

**FIGURE 1:**
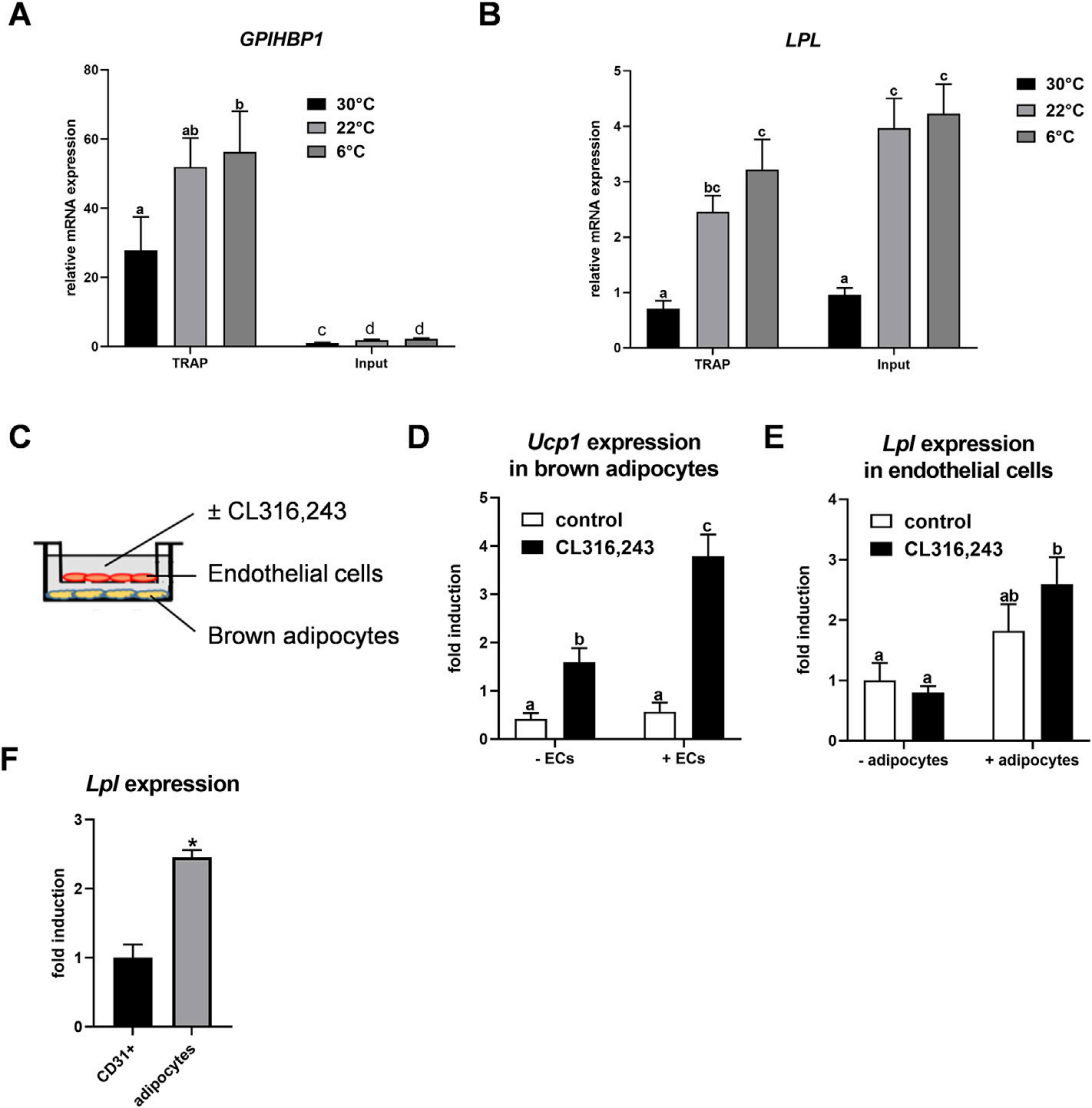
Endothelial expression of LPL in BAT. **(A–B)** EndoNuTRAP mice were housed at 22°C, at 6°C or at 30°C for 3d. Expression of *Gpihbp1* **(A)** and *Lpl* **(B)** in endothelial cells (TRAP) and total tissue (input) of BAT were analyzed by qPCR (n = 4–5). **(C)** Transwell system for co-culturing of murine brown adipocytes with murine microvascular endothelial cells. Endothelial cells were cultured in the presence or absence of differentiated brown adipocytes and were incubated without or with the β3-adrenergic agonist CL316,243 at the same time to thermogenically activate the brown adipocytes. **(D)** *Ucp1* expression in brown adipocytes co-cultured without and with endothelial cells (ECs) (n = 4). **(E)** *Lpl* expression in endothelial cells co-cultured with brown adipocytes (+adipocytes) or without (-adipocytes), stimulated with CL316,243 or with vehicle (none) (n = 4). (F) Wild type mice were housed at 6°C for 3 days and expression of *Lpl* was determined in CD31-positive endothelial cells and thermogenic adipocytes of BAT that were isolated by MACS^®^ (n = 3)Results are presented as mean values ±SEM. Statistical significance for **(A,B)** was determined by one-way ANOVA and for **(D,E)** by two-way ANOVA. Same letter denotes groups that are not significantly different from each other (*p* ≥ 0.05). Statistical significance for **(F)** was determined by Student’s t test; *p < 0.05.

Previously we described that *Lpl* is expressed in CD31-positive endothelial cells of BAT (Fischer et al., 2021). To confirm this finding, we employed NUTRAP mice, a Cre recombinase-dependent transgenic model that allows cell type-specific gene expression analysis by translating ribosome affinity purification (TRAP). This method employs pulldown of polyribosomes and thus does not require tissue disintegration (Roh et al., 2017). Endothelium-specific NuTRAP (EndoNuTRAP) mice were generated by crossing NuTRAP with VE-cadherin (Cdh5)-Cre mice. To analyze the temperature-dependence of *Lpl* expression, EndoNuTRAP mice were kept at room temperature (22 °C), or exposed to either cold (6°C) or 30°C for 3 days. Efficient enrichment of mRNA from endothelial cells in the BAT lysates was confirmed by high expression of the endothelial marker *Gpihbp1* in the pulldown (TRAP) fraction as compared to whole tissue lysate (input) at all temperatures (Figure 1A). Of note, we observed substantial expression of *Lpl* in the endothelial cell fraction that increased with decreasing housing temperatures of the EndoNuTRAP mice (Figure 1B). To further investigate the expression of *Lpl* in endothelial cells, we established a transwell system for co-culturing murine microvascular endothelial cells with murine brown adipocytes (Figure 1C). To evaluate the potential mutual effects on gene expression, we cultured cells alone or in combination that were treated without or with the β3-adrenergic agonist CL316,243. *Ucp1* expression was induced in CL316,243-treated brown adipocytes (Figure 1D), which confirms their thermogenic activation. Endothelial cells co-cultured with quiescent brown adipocytes showed a trend for increased *Lpl* expression in comparison to controls without adipocytes (Figure 1E). This effect was significant after additional incubation with the ß3-adrenergic receptor agonist CL316,243 (Figure 1E). These results suggested that activated brown adipocytes produce paracrine signals that lead to increased *Lpl* expression in endothelial cells. To estimate *Lpl* expression in endothelial cells versus adipocytes from BAT of cold-exposed mice, we employed antibody-based magnetic-activated cell sorting (Fischer et al., 2021). In this setup, *Lpl* mRNA levels were approximately 2.5-fold higher in thermogenic adipocytes compared endothelial cells (Figure 1F). Taken together, *Lpl* is expressed at appreciable levels in endothelial cells of cold-activated BAT and endothelial *Lpl* expression is stimulated by brown adipocytes.

### Effect of Endothelial Cell Specific LPL Knockout on Energy Homeostasis

**FIGURE 2:**
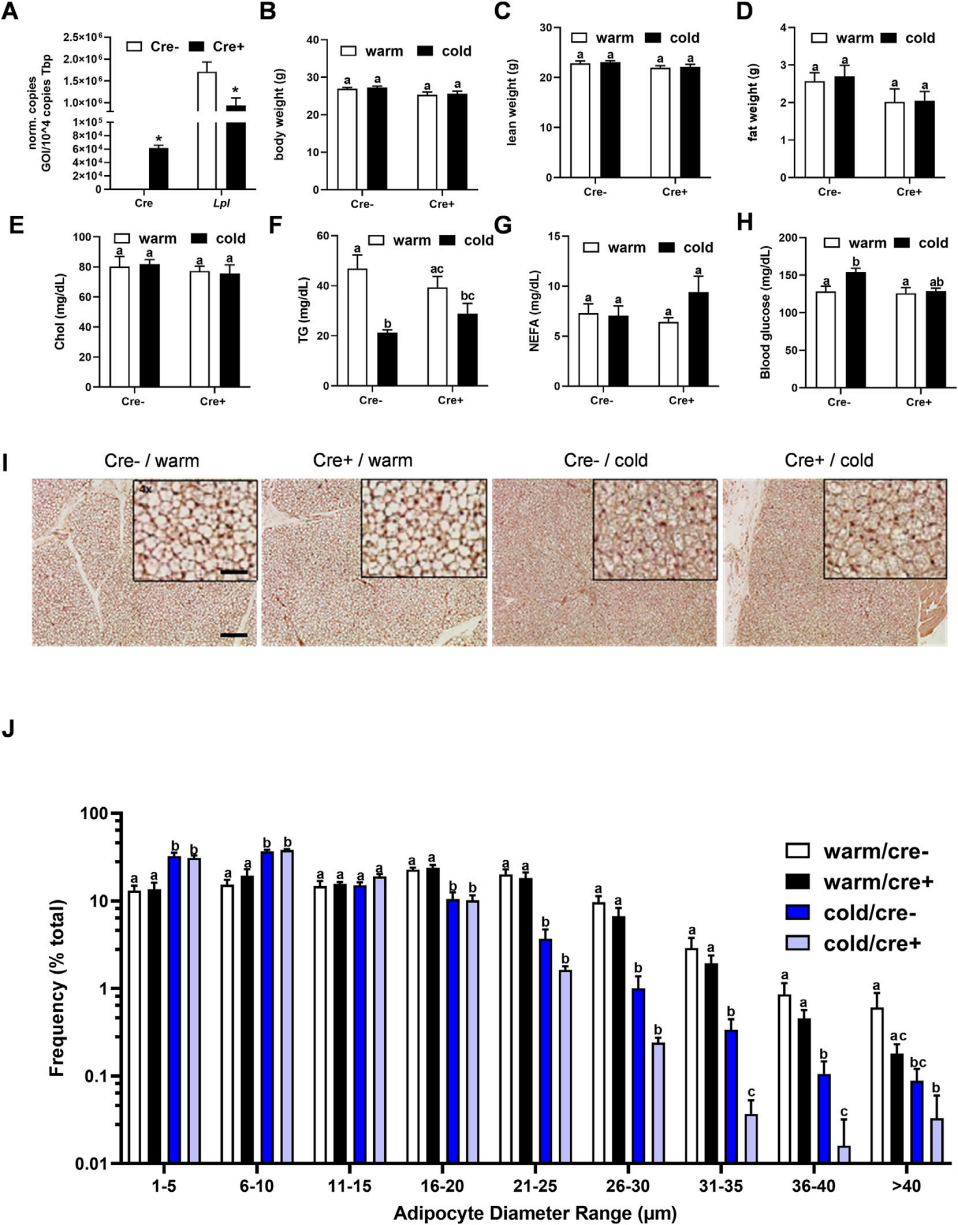
Body composition and BAT lipid content in endothelial cell specific LPL knockout mice. **(A)** Control and EndoLPLko mice received a tamoxifen dosage to generate mice lacking LPL in endothelial cells (Cre+) and controls (Cre-). Mice were exposed to 6°C for 1 day before SVF was isolated from BAT and analyzed for Cre and Lpl expression by qPCR (n = 4). **(B–E)** EndoLPLko mice and litter mates (n = 6) were fed a western-type diet for 2 weeks, and housed at 6°C (cold) or 30°C (warm) in the second week. **(B)** Body weight, **(C)** lean weight and **(D)** fat weight determined by EchoMRI, **(E)** plasma cholesterol (chol), **(F)** triglycerides (TG), **(G)** non-esterified fatty acids (NEFA) and **(H)** blood glucose. **(I)** Hematoxylin eosin staining of BAT. Bars indicate 200 and 50 µm for lower and higher magnification, respectively. **(J)** Ranges of adipocyte diameters calculated from **(I)** using NIS-Elements (Nikon^®^). Results are presented as mean values ±SEM. Statistical significance was determined by Student’s *t* test **(A)** or by two-way ANOVA **(B–D)**. Same letter denotes groups that are not significantly different from each other (*p* ≥ 0.05).

To analyze the relevance of endothelial LPL for BAT energy metabolism, endothelial specific LPL knockout mice were created by crossing mice expressing a tamoxifen-inducible Cre recombinase under the control of the VE-cadherin (Cdh5) promoter with floxed *Lpl* mice to generate mice lacking LPL in endothelial cells (EndoLPLko) and control littermates. Cre recombinase expression was detected in stromal vascular fraction (SVF) isolated from BAT of EndoLPLko but not in control mice (Figure 2A). Moreover, significant reduced expression of *Lpl* in the SVF confirmed efficient knockdown (Figure 2A). Next, we assessed the effect of the endothelial cell-specific knockout on energy balance and metabolism. To create nutritional conditions typical for humans that go along with elevated lipoprotein turnover in adipose tissues (Worthmann et al., 2017), the study was performed with mice that were fed western type diet for 2 weeks. Mice were then housed at thermoneutrality (30°C), a state of thermogenic inactivity (Fischer et al., 2018), or at 6°C (cold) to strongly activate BAT. Under these conditions, body weight (Figure 2B), lean weight (Figure 2C), fat weight (Figure 2D), plasma cholesterol (Figure 2E), triglycerides (Figure 2F), non-esterified fatty acids (Figure 2G) and blood glucose (Figure 2H) were not different in EndoLPLko mice compared to control littermates. Except for plasma triglycerides, no significant effects of housing temperature on these parameters was observed. Furthermore, lack of LPL in endothelial cells had no effect on histological BAT appearance studied by hematoxylin and eosin staining (Figure 2I). As expected, the diameter of brown adipocytes was smaller in cold-exposed compared to thermoneutral-housed mice but we did not observe a major effect by the genotype (Figure 2J).

**FIGURE 3:**
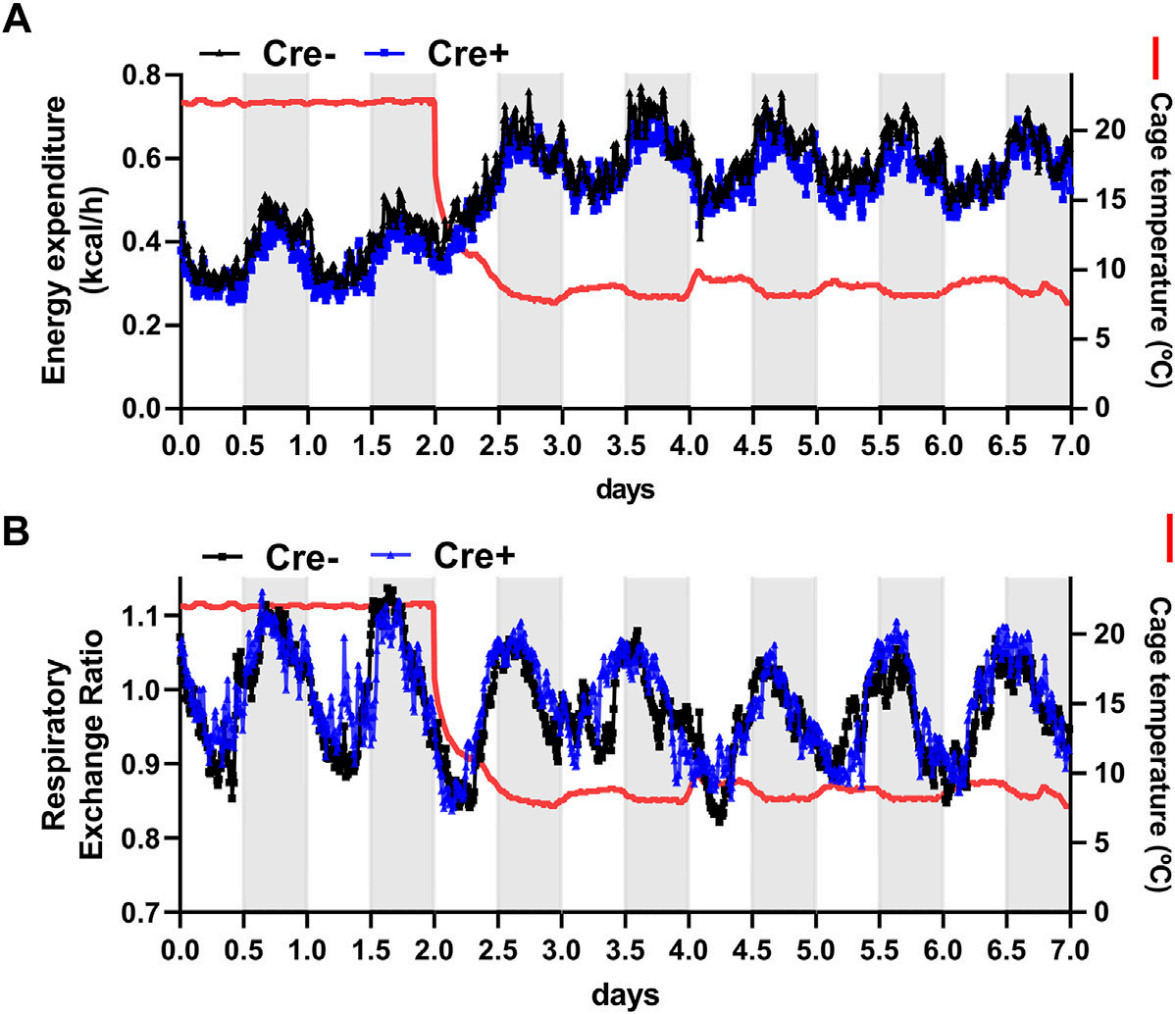
Energy expenditure of endothelial cell-specific LPL knockout mice. For indirect calorimetry analysis EndoLPLko (Cre+) and control (Cre-) mice fed a western-type diet were placed in a TSE phenomaster system. **(A)** Energy expenditure and **(B)** respiratory exchange ratio were determined by indirect calorimetry (n = 3–4). Results are presented as mean values.

To further address the role of endothelial cell-specific LPL expression for whole body energy balance in adaptation to cold ambient temperature, EndoLPLko and control mice were subjected to indirect calorimetry. Energy expenditure increased in both genotypes when the mice were exposed to cold temperature (Figure 3A). Furthermore, respiratory exchange rate was slightly lower under these conditions, indicating higher lipid compared to glucose oxidation (Figure 3B). However, the EndoLPLko mice exhibited no significant difference in energy expenditure or respiratory exchange rate compared to the Cre-controls, indicating preserved systemic energy homeostasis in the absence of endothelial LPL.

### Organ Energy Uptake, Plasma Lipids and BAT Gene Expression in the Postprandial State

**FIGURE 4:**
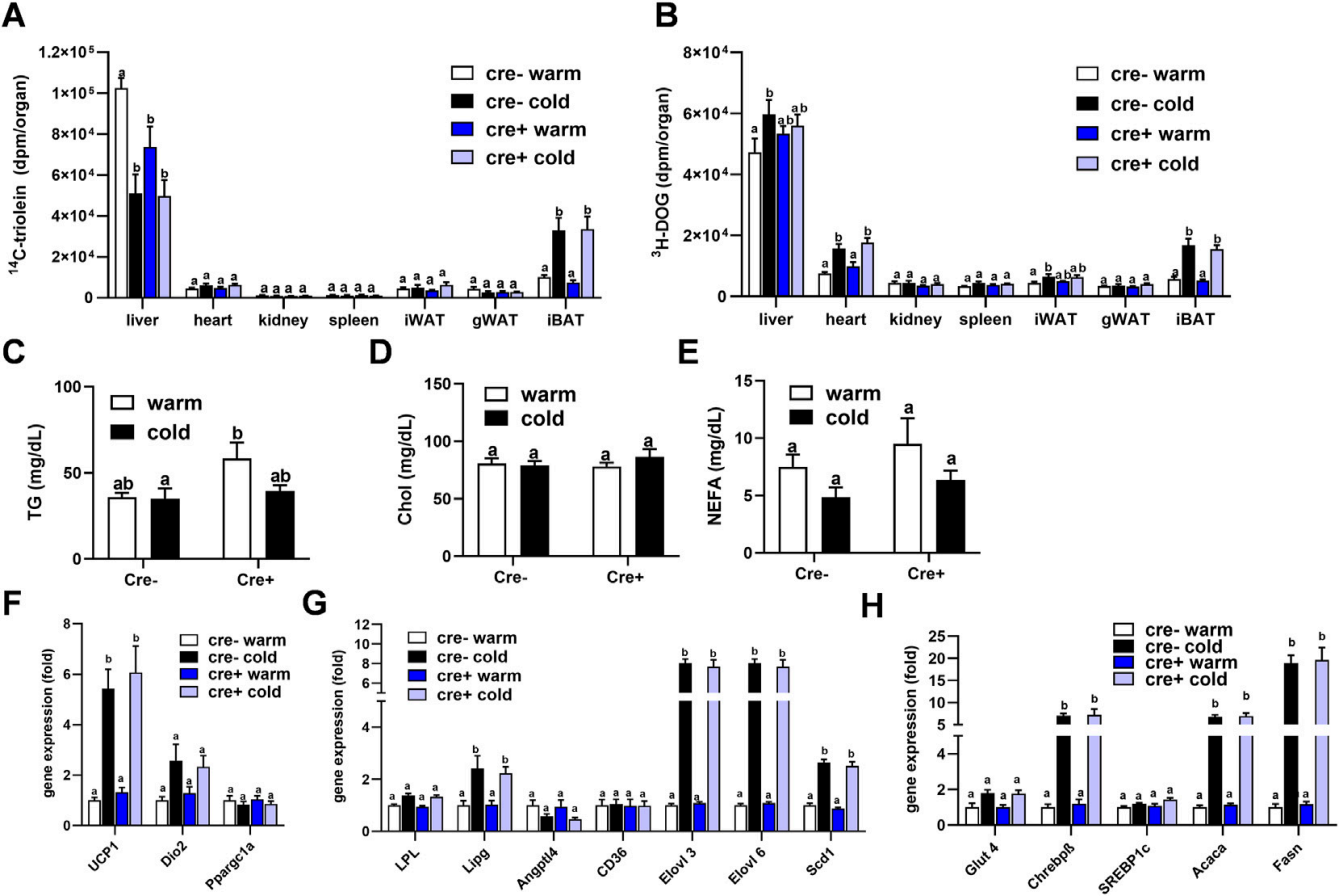
Organ lipid and glucose uptake and BAT gene expression in the postprandial state. EndoLPLko (Cre+) mice and Cre-litter mates (n = 6) were fed a western-type diet for 2 weeks, and housed at 6°C (cold) or 30°C (warm) in the second week. Organ and plasma harvest was performed 2 h after a combined oral glucose and fat gavage. **(A)** Organ uptake of ^14^C-Triolein and **(B)** ^3^H-deoxyglucose (^3^H-DOG). Plasma levels of **(C)** triglycerides, **(D)** cholesterol and **(E)** Non-esterified fatty acids (NEFA). Expression of **(F)** thermogenic, **(G)** lipoprotein and lipid-handling, and **(H)** glucose-handling genes in BAT of EndoLPLko mice. Results are presented as mean values ±SEM. Statistical significance was determined by two-way ANOVA (*p* < 0.05). Same letter denotes groups that are not significantly different from each other (*p* ≥ 0.05).

To study whether lack of endothelial LPL affects organ-specific energy handling in the postprandial state, EndoLPLko and control mice kept on a western-type diet received a combined fat and glucose gavage containing ^3^H-deoxyglucose (^3^H-DOG) and ^14^C-triolein as radiolabeled tracers. In line with previous data (Bartelt et al., 2011), uptake of ^14^C-triolein into BAT was increased in cold-exposed mice whereas lipid uptake into the liver was diminished (Figure 4A). No significant differences were detected in the other organs investigated. Of note, no effect of endothelial-specific LPL knockdown on ^14^C-triolein uptake in any organ could be detected, except for a significantly lower hepatic lipid uptake in warm-housed mice (Figure 4A). As expected, ^3^H-DOG uptake into BAT was higher in cold-exposed mice, and this was additionally observed for heart, inguinal WAT and liver (Figure 4B). Again, no effect of the different genotypes was observed. Plasma analysis demonstrated that the concentrations of triglycerides (Figure 4C), cholesterol (Figure 4D) and non-esterified fatty acids (Figure 4E) in these mice were not different between the experimental groups.

These data indicate that TRL and glucose disposal in BAT is independent of endothelial LPL expression in the postprandial state. Next, we tested whether this, and the lacking effect of endothelial LPL knockout on energy homeostasis (Figure 3), might be due to compensation by altered BAT expression of genes critical for thermogenesis (Figure 4F), lipoprotein and lipid processing (Figure 4G) or glucose handling (Figure 4H). As predicted, cold exposure led to the induction of *Ucp1* (Figure 4F) (Cannon and Nedergaard, 2004), endothelial lipase (*Lipg*) (Schaltenberg et al., 2021) and fatty acid elongase 3 (*Elovl3*) (Jakobsson et al., 2005) (Figure 4G). Also in line with previous work (Sanchez-Gurmaches et al., 2018), glucose-regulated DNL genes including carbohydrate response element-binding protein-β (*Chrebpβ*), *Elovl6*, acetyl-CoA carboxylase-α (*Acaca*) and fatty acid synthase (*Fasn*) were induced by cold (Figures 4G,H). However, at a given housing temperature expression of these genes was not different in EndoLPLko mice compared to littermates, suggesting that endothelial LPL does not influence postprandial BAT energy and lipid homeostasis.

### Lipoprotein Disposal and BAT Gene Expression in the Fasted State

**FIGURE 5:**
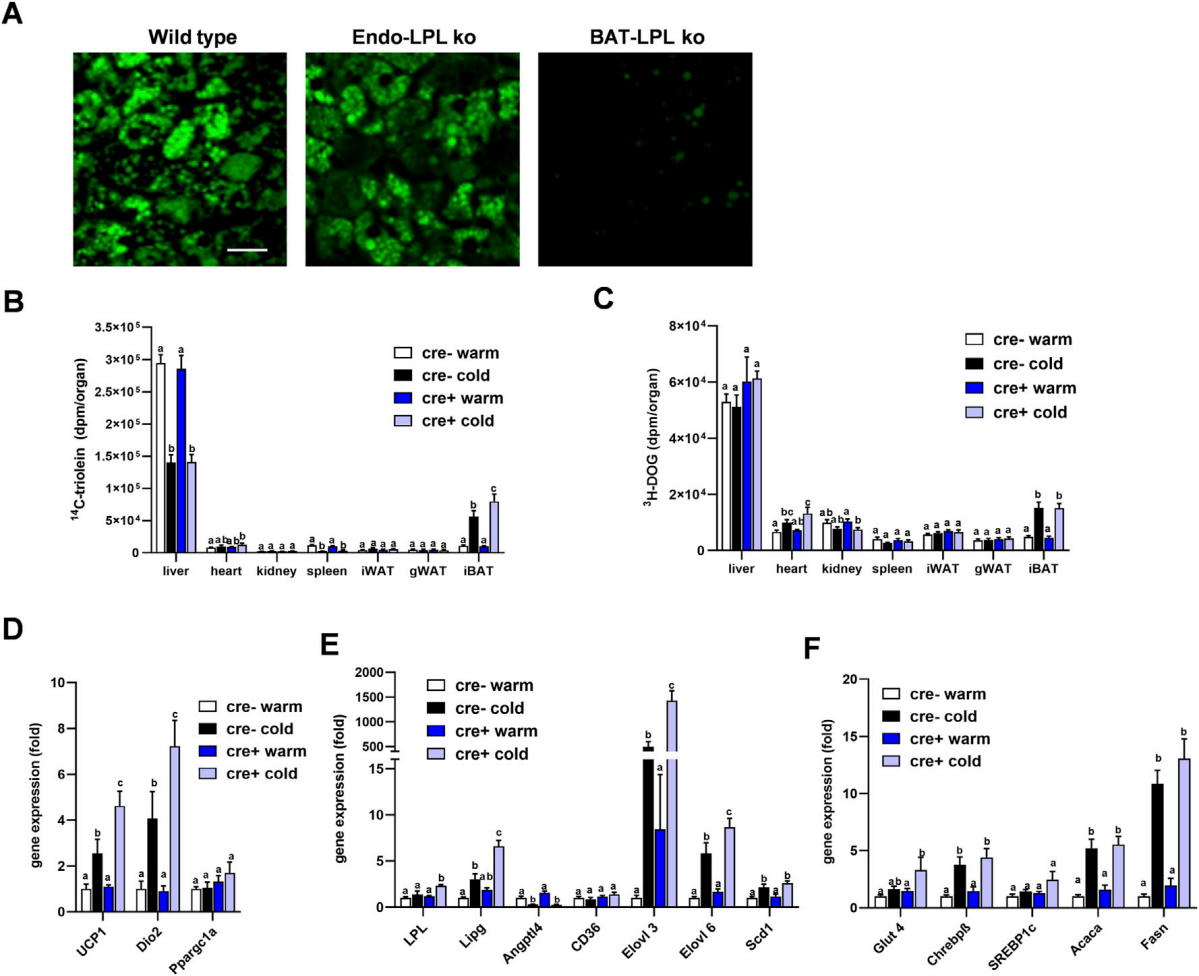
Lipoprotein disposal and BAT gene expression in the fasted state. **(A)** EndoLPLko, mice lacking LPL in brown adipocytes (BATLPLko), and wild type control mice fed a chow diet were housed at 22°C followed by cold exposure (6°C) for 24 h. To analyze TRL uptake, BODIPY-labeled TRL were injected intravenously into animals fasted for 4 h. BAT was harvested for *ex vivo* confocal fluorescence microscopy 15 min after injection. The figure shows representative images of TRL uptake. Bar indicates 20 µm. **(B–F)** EndoLPLko and control mice (n = 5–6) were fed a western-type diet for 2 weeks and housed at 30°C (warm) or 6°C (cold) in the second week. ^14^C-Triolein labeled TRLs were injected intravenously together with tracer amounts of ^3^H-DOG. Organs were harvested 15 min after injection. **(B)** Organ uptake of ^14^C-triolein, and **(C)** ^3^H-DOG in EndoLPLko (Cre+) and control (Cre-) mice. Gene expression of **(D)** thermogenic, **(E)** lipoprotein and lipid-handling, and **(F)** glucose-handling genes in BAT of EndoLPLko and Cre-control mice. Values are shown relative to Cre-warm. Results are presented as mean values ±SEM. Statistical significance was determined by two-way ANOVA. Same letter denotes groups that are not significantly different from each other (*p* ≥ 0.05).

Insulin action on brown adipocytes has been shown to increase LPL-dependent lipoprotein disposal in BAT (Heine et al., 2018), and thus high insulin levels in the postprandial state may mask potential effects of LPL produced in endothelial cells. To investigate the role of endothelial cell LPL in BAT lipid disposal under low insulin conditions, we intravenously injected fluorescently-labeled TRL into mice fasted for 4 h. Interscapular BAT was studied *ex vivo* by confocal microscopy 15 min after the injection. Brown adipocytes from control animals (wild type) showed a strong accumulation of BODIPY-labelled fatty acids within their lipid droplets and the same was observed for EndoLPLko mice (Figure 5A). In contrast, mice lacking LPL selectively in brown adipocyte (BAT-LPLko) showed little accumulation of BODIPY fatty acids in BAT. Thus, LPL in endothelial cells, unlike that in brown adipocytes, is not essential for uptake of TRL-derived fatty acids in the fasted state. To further address this notion in a more quantitative fashion, we intravenously injected TRL labeled with ^14^C-triolein together with ^3^H-DOG in tracer amounts into fasted mice that were kept on a western type diet and either housed at 30°C (warm) or at 6°C (cold). Notably, we observed that the cold-dependent increase in BAT ^14^C-triolein uptake was moderately but significantly higher in EndoLPLko mice compared to controls (Figure 5B). In contrast, other organs exhibited no genotype effect. In contrast to lipid uptake, ^3^H-DOG uptake into BAT was not influenced by the absence of LPL in endothelial cells (Figure 5C). Of note, further BAT analysis revealed that cold-exposed EndoLPLko mice compared to controls exhibited increased expression of the thermogenic markers *Ucp1* and deiodinase-2 (*Dio2*) (Figure 5D), and of the lipases *Lpl*, *Lipg* as well as the fatty acid elongases *Elovl3* and *Elovl6* (Figure 5E). In line with the lack of genotype effect on glucose handling, no effect was observed for glucose-handling genes including DNL enzymes in cold-exposed EndoLPLko versus control mice (Figure 5F). Altogether, the lack of endothelial LPL resulted in higher lipid disposal by activated BAT, which may be explained by the compensatory induction in the expression of adipocyte LPL and endothelial LIPG.

## Discussion

Activated BAT is characterized by efficient, LPL-dependent disposal of TRL, a metabolic action that is pronounced enough to reverse pathological hypertriglyceridemia (Bartelt et al., 2011). While it was originally assumed that expression of the enzyme in adipose tissue occurs mainly in adipocytes and macrophages, in the present study we detected substantial expression of LPL in endothelial cells of cold-activated BAT. To address the functional role of LPL produced by these cells, we generated and analyzed endothelium-specific LPL knockout (EndoLPLko) mice. Notably, we observed that uptake of fatty acids from TRL is not diminished in either the postprandial or the fasted state. This indicates that LPL produced in endothelial cells is not rate-limiting for TRL triglyceride hydrolysis, despite its close proximity to the site of lipoprotein processing on the vascular face of the endothelium (Goulbourne et al., 2014). Previous work showed that lack of TRL processing in BAT and WAT results in induction of glucose uptake and compensatory *de novo* synthesis of fatty acids (Weinstock et al., 1997; Ullrich et al., 2001; Bartelt et al., 2013). In the present study, we found no effect on BAT glucose uptake and unaltered, glucose-dependent DNL enzyme expression in EndoLPLko mice, indirectly supporting the notion, that influx of lipids into the organ is not altered in a meaningful manner. The underlying reason may be that endothelial LPL is quantitatively irrelevant compared to LPL produced by brown adipocytes that represent the majority of metabolic activity in BAT. LPL produced by activated brown adipocytes controls BAT TRL disposal, as shown in the present study using brown adipocyte-specific knockout, and previously using adipocyte-specific knockouts (Bartelt et al., 2013). Another reason might be, that LPL secretion is less efficient in endothelial cells than in adipocytes, which has been shown to be sensitive to intracellular disturbances requiring specific chaperones and tight redox regulation (Kristensen et al., 2021).

Apart from the lack of direct contribution to TRL hydrolysis, we provide clear evidence that endothelial LPL has a regulatory function in BAT TRL disposal. This is demonstrated by the significant induction of *Lpl* and *Lipg* in BAT of fasted EndoLPLko mice. This unexpected finding suggests that, normally, endothelial LPL generates specific autocrine and paracrine signals to suppress the lipases LIPG and LPL. Consistent with such mechanisms, induction of *Lipg* has previously been observed in mice lacking *Lpl* in whole adipose tissues (Kratky et al., 2005) but not in mice lacking *Lpl* only in adipocytes (Bartelt et al., 2013). Thus, endothelial LPL appears to have specific regulatory functions. This notion is underlined by our observation that not only lipases but also *Ucp1*, *Dio2*, *Elovl3* and *Elovl6* are upregulated in BAT of the fasted EndoLPLko mice, indicating that the genes of these thermogenic and fatty acid-processing markers are under control of endothelial LPL. The mechanisms underlying this regulation remain elusive but it is tempting to speculate that LPL, which has no known function as gene regulator, may affect gene expression via a fatty acid-related transcriptional mechanism. Supporting that view, it was recently shown that intracellular lipolysis mediated by lysosomal acid lipase and subsequent fatty acid oxidation in capillary endothelial cells of BAT is required for activation of the angiogenic transcription hypoxia induced factor (Fischer et al., 2021). This process is important for proper endothelial proliferation, vascularization and thermogenic function of BAT and WAT in response to cold exposure (Fischer et al., 2021; Fischer et al., 2022).

In conclusion, we found that LPL is expressed by endothelial cells of cold-activated BAT. Interestingly, endothelial LPL activity seems not to be involved in lipoprotein clearance needed for energy replenishment but appears to fine-tune the metabolic balance by generating fatty acid-dependent signals that modulate transcriptional pathways in BAT in response to cold exposure.

## Data Availability

The original contributions presented in the study are included in the article/Supplementary Material, further inquiries can be directed to the corresponding author.
